# Failure of γδ T Cell Recovery in the Gut With Effective Anti‐HIV Therapy

**DOI:** 10.1155/jimr/9883892

**Published:** 2025-11-27

**Authors:** Priscila O. Barros, Stephanie C. Burke Schinkel, Ameeta Lubina Nayak, Brittany Haas, Tamara K. Berthoud, Michaeline McGuinty, D. William Cameron, Jonathan B. Angel

**Affiliations:** ^1^ Inflammation and Chronic Disease Program, Ottawa Hospital Research Institute, Ottawa, Ontario, Canada, ohri.ca; ^2^ Department of Biochemistry, Microbiology and Immunology, University of Ottawa, Ottawa, Ontario, Canada, uottawa.ca; ^3^ Department of Medicine, Division of Infectious Diseases, The Ottawa Hospital, University of Ottawa, Ottawa, Ontario, Canada, uottawa.ca

**Keywords:** alpha 4 beta 7 integrin (α4β7), cytokine polyfunctionality, γδ T cells, human immunodeficiency virus (HIV), recto-sigmoid colon biopsy

## Abstract

**Introduction:**

Infection with HIV alters γδ T cells, and while these changes have been well documented in blood, they are less well understood in the gut.

**Methods:**

Phenotype (specifically HIV coreceptor expression) and polyfunctionality, as defined by concomitant cytokine expression, were evaluated in γδ T cells in the blood and gut of effectively treated people living with HIV (PLWH) and people without HIV (PWOH). Mononuclear cells were isolated from blood and recto‐sigmoid colon biopsy tissue, and γδ T cells were evaluated by flow cytometry.

**Results:**

The expression of α4β7 was significantly higher in gut‐derived γδ T cells of PLWH compared to PWOH and blood‐derived cells of both groups. The polyfunctionality profile of gut‐derived γδ T cells in PLWH was also different than that of PWOH, with significantly higher expression of IFN‐γ in the gut‐derived cells.

**Conclusion:**

The alterations observed in gut‐derived γδ T cells from effectively treated PLWH suggest cellular function is not restored with prolonged antiretroviral therapy (ART) and may contribute to a chronic inflammatory state.

**Trial Registration:** HAVARTI trial registration (ClinicalTrials.gov): NCT03147859

## 1. Introduction

γδ T cells are considered unconventional T cells with properties of both innate and adaptive immunity. In humans, most γδ T cells express either Vδ1 or Vδ2 chains; Vδ1 is generally present in higher proportions in the gut, skin, and liver, whereas Vδ2 cells are more prevalent in the peripheral blood [1]. γδ T cells can be cytotoxic effectors and express various cytotoxicity‐related markers such as CD8, CD69, CD56^2^ [2], and NKG2D [1], and can rapidly produce large amounts of proinflammatory cytokines, including IL‐17, TNF‐α, and IFN‐γ [3, 4].

In HIV infection, an inversion of the Vδ2:Vδ1 ratio has been extensively reported in peripheral blood [5–9]. The inversion has been attributed to expansion of the Vδ1 population and to depletion of Vδ2 cells [6]. The Vδ2 T cell subset was shown to be permissive to HIV infection, due in part to expression of α4β7 and high levels of CCR5, two known HIV coreceptors. Moreover, impairment of blood‐derived Vδ2 T cell function (i.e., reduction of antigen‐induced cytokine production and proliferative/cytotoxic capacity) has also been reported during HIV infection [4, 8, 10, 11]. These mechanisms have been suggested to play a role in the inversion of the Vδ2:Vδ1 ratio [12, 13], and these cells may be early targets of HIV replication [14]. The inversion in Vδ2:Vδ1 ratio is also observed in the gastrointestinal tract of people living with HIV (PLWH), where γδ T cells are one of the first lines of defense, and while highly active antiretroviral therapy (ART) lowers the percent of γδ+ CD3+ T cells to similar levels seen in people without HIV (PWOH), the change in ratio persists [15]. The function and phenotype of gut‐derived γδ T cells is less well known, though studies have shown that infection with HIV leads Vδ1 to shift their memory phenotype and lose CD45RA expression, and Vδ2 to produce less IFN‐γ [5] while γδ T cells in general show an increase in the gut‐homing markers CCR9 or CD103[15].

In the present study, γδ T cell polyfunctionality, as defined by concomitant cytokine expression, was evaluated in the blood and gut of effectively treated PLWH and PWOH. Our results demonstrate that gut‐derived γδ T cells skew toward a more Th1‐like phenotype, characterized by significantly higher expression of IFN‐γ, which suggests that despite prolonged suppressive ART, the impact of HIV on the function of gut‐derived γδ T cells persists. The differences reported here may be due to alterations in the gut barrier and the development of chronic immune activation, which are hallmarks of HIV infection.

## 2. Materials and Methods

### 2.1. Study Participants, Sample Collection, and Cell Isolation

Blood and recto‐sigmoid colon biopsy samples were collected from PLWH participating in a clinical trial [16] at their baseline visit while on effective ART. Blood was also collected from PWOH. Gut biopsies from PWOH were collected during routine screening colonoscopies where there were no documented comorbidities, and no sign of disease was observed during the procedure. Table 1 for participant characteristics (mean ± SD of age, gender, duration on ART below detection (years), CD4 count, and CD4:CD8 ratio are shown in bold, as applicable). All participants provided written informed consent, as per study approval by the Ottawa Health Science Network Research Ethics Board (OHSN‐REB 20160928 and 2005256‐01H) (Ottawa, ON, Canada).

**Table 1:** Participant characteristics.

Age	Gender	Duration on ART below detection (years)	CD4 count	CD4:CD8 ratio
PLWH				
61	Male	4	619	0.61
27	Male	3	1020	1.21
37	Male	5	587	0.85
34	Male	4	828	1.24
34	Male	5	729	1.06
38	Male	4	892	0.79
46	Male	6	676	1.02
36	Male	7	805	1.7
29	Male	6	662	1.28
**38.00 ± 10.2**	**9 males**	**4.89 ± 1.27 years**	**757.6 ± 141**	**1.08 ± 0.32**
PWOH (PBMC)				
33	Female	—	—	—
47	Female	—	—	—
53	Female	—	—	—
30	Male	—	—	—
23	Male	—	—	—
59	Male	—	—	—
56	Male	—	—	—
38	Female	—	—	—
41	Male	—	—	—
**42.22 ± 12.42**	**5 males:4 females**	—	—	—
PWOH (RMC)				
80	Male	—	—	—
65	Male	—	—	—
58	Female	—	—	—
55	Male	—	—	—
68	Female	—	—	—
60	Male	—	—	—
64	Male	—	—	—
63	Male	—	—	—
**64.13 ± 7.62**	**6 males:2 females**	—	—	—

Peripheral blood mononuclear cells (PBMCs) were isolated from heparinized whole blood by density gradient centrifugation using Lymphoprep (Stemcell, Vancouver, Canada), as described previously [17] and cultured in RPMI‐1640 media supplemented with 0.25 mM L‐glutamine, 100 μg/mL penicillin–streptomycin, and 10% fetal bovine serum (FBS) (Gibco).

Recto‐sigmoid colon pinch biopsies were obtained from 8 to 12 different areas in the recto‐sigmoid colon. Recto‐sigmoid mononuclear cells (RMC) were isolated by enzyme digestion, as described previously [17]. The collected tissues were minced with a scalpel, incubated for 2 h at 225 rpm and 37°C in RMC media (RPMI supplemented with 10% FBS, 1% HEPES, 100 μg/mL penicillin–streptomycin (Gibco), and 0.1 mg/mL gentamicin (Sigma)) with collagenase type IV (1000iU, Gibco), DNase1 (10U, Sigma), and Tazocin (2.3 mg piperacillin, 0.29 g tazobactam; Sandoz, Basel, Switzerland), followed by needle homogenization with 18 then 20 gauge needles. Isolated cells were cultured in RMC media.

### 2.2. Flow Cytometry

To evaluate the expression of cell surface markers and cytokines by γδ T cells, intracellular cytokine staining was performed on unstimulated or stimulated cells. For this, PBMC or RMC were stimulated with PMA (0.081 µM) and Ionomycin (1.34 µM) (500x Cell activation cocktail, Biolegend), Brefeldin A (10 μg/mL, Sigma), and GolgiStop (0.5μL/mL, BD Biosciences) for 5 h. Cells were stained and fixed with the BD Cytofix/Cytoperm kit (BD Biosciences). Flow cytometry stains and antibodies used included LIVE/DEAD Fixable Aqua Dead Cell Stain Kit (Life Technologies Cat# L34957), CD45‐APC/Cy7 (BioLegend Cat# 368516, RRID: AB_2566376), CD4‐Brilliant violet 650 (BioLegend Cat# 317436, RRID: AB_2563050), CD3‐Alexa fluor 700 (BioLegend Cat# 300424, RRID: AB_493741), CD8‐Brilliant Violet 711 (BioLegend Cat# 344734, RRID: AB_2565243), CCR5‐Alexa Fluor 488 (BioLegend Cat# 359104, RRID: AB_2562313), TCR γ/δ‐PE/Dazzle 594 (BioLegend Cat# 331226, RRID: AB_2565534), IL‐22‐PerCP/Cy5.5 (BioLegend Cat# 366710, RRID: AB_2566794), IFN‐γ‐Pac Blue (BioLegend Cat# 502522, RRID: AB_893525), TNF‐α‐Brilliant Violet 785 (BioLegend Cat# 502948, RRID: AB_2565858) (Biolegend), IL‐17A‐PE (Thermo Fisher Scientific Cat# 12‐7179‐42, RRID: AB_1724136) and alpha4/beta7 [A4B7R1]‐APC (engineered and produced by the Nonhuman Primate Reagent Resource (NIH Nonhuman Primate Reagent Resource Cat#PR‐1421, RRID: AB_2819257).

Cells were analyzed on a BD–LSR Fortessa flow cytometer (BD Biosciences, Heidelberg, Germany (RRID: SCR_025285)) and data acquired with BD FACSDiva software v8.0 (BD Biosciences, San Jose, CA, USA BD (RRID: SCR_001456)). The values detected in the cultures without stimulation (background) were subtracted from the stimulated ones, and positive gates were set by fluorescence minus one (FMO) or negative staining controls.

### 2.3. Data Analysis

Data analysis and Boolean combination gating were performed in FlowJo v10.7 (FlowJo LLC, Ashland, OR, USA [RRID: SCR_008520]). Graphs were created using GraphPad Prism v9.4.1 (GraphPad Software Inc., San Diego, CA, USA [RRID: SCR_002798]). Pie charts for polyfunctionality and permutation tests for statistical differences were created with NIH SPICE (Simplified Presentation of Incredibly Complex Evaluations) v6.1 (NIH NIAID, Bethesda, MD, USA [RRID: SCR_016603])[18]. Polyfunctionality was defined as the percentage of γδ T cells expressing more than one cytokine evaluated in the study (IL‐17, TNF‐α, and IFN‐γ). For the statistical analysis of differences between groups, ANOVA and Tukey’s test were performed. Data are shown as mean ± SD. Differences were considered significant when *p* ≤ 0.05.

## 3. Results

### 3.1. Baseline Proportion of γδ T Cells and Their Expression of CCR5, α4β7, and CD8

The proportion of γδ T cells within the total T cell population was evaluated, both in peripheral blood (PBMC) and in the recto‐sigmoid colon (RMC), as well as expression of cell surface markers of interest. Significantly higher proportions of γδ T cells were observed in the gut biopsies of both PLWH and PWOH, when compared to the prevalence in peripheral blood (Figure 1A).

Figure 1Analysis of γδ T cells in PBMC and RMC from ART‐treated PLWH and PWOH. PBMC and RMC were isolated from PWOH (HIV−) and PLWH (HIV+), and γδ T cells were assessed by flow cytometry. (A) Proportion of γδ T cells in the CD3+ T cell population, γδ T cell expression of (B) CCR5, (C) α4β7, and (D) CD8 was evaluated without stimulation (PBMC: HIV− *n* = 9, HIV+ *n* = 8; RMC: HIV− *n* = 8, HIV+ *n* = 6). (E) Gating strategy used for both PBMC and RMC (shown), and representative dot plots for γδ, CCR5, α4β7, and CD8 expression are provided for one PLWH donor, in RMC and PBMC. Significant differences are shown in the graph, calculated by ANOVA and Tukey’s test.

**Figure:**
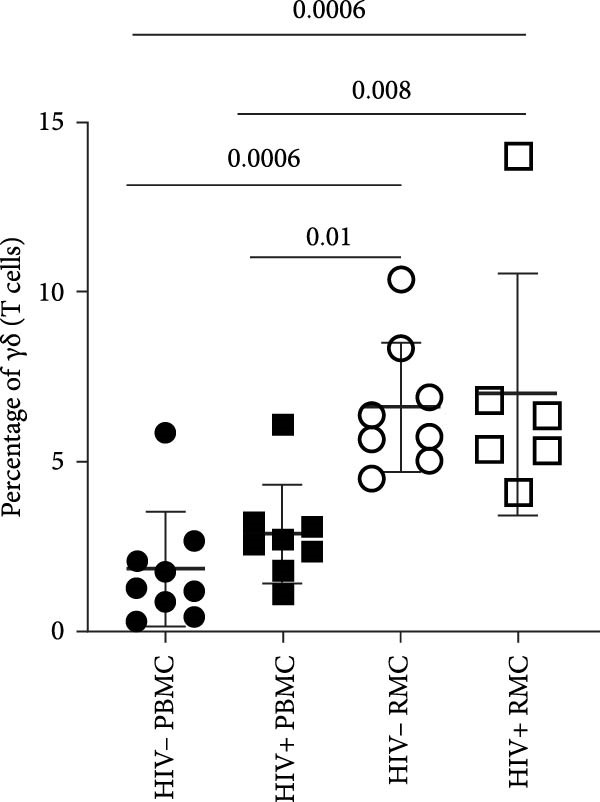
(A)

**Figure:**
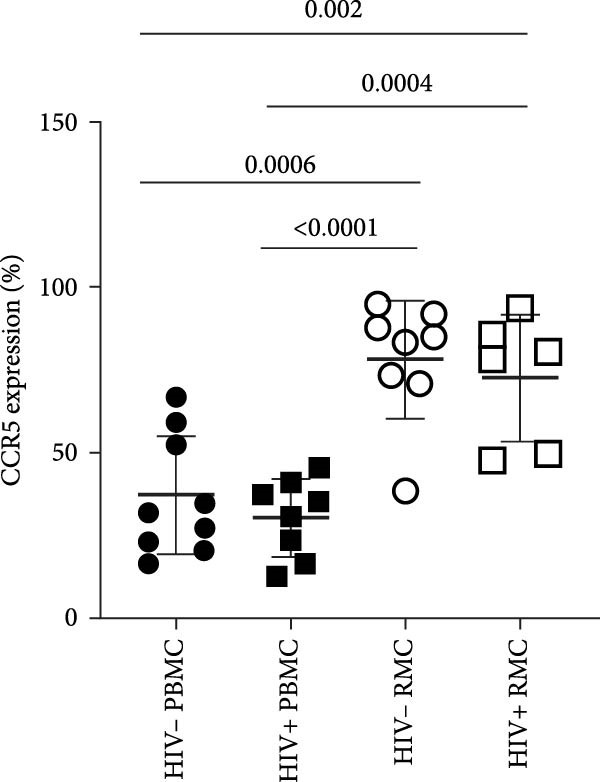
(B)

**Figure:**
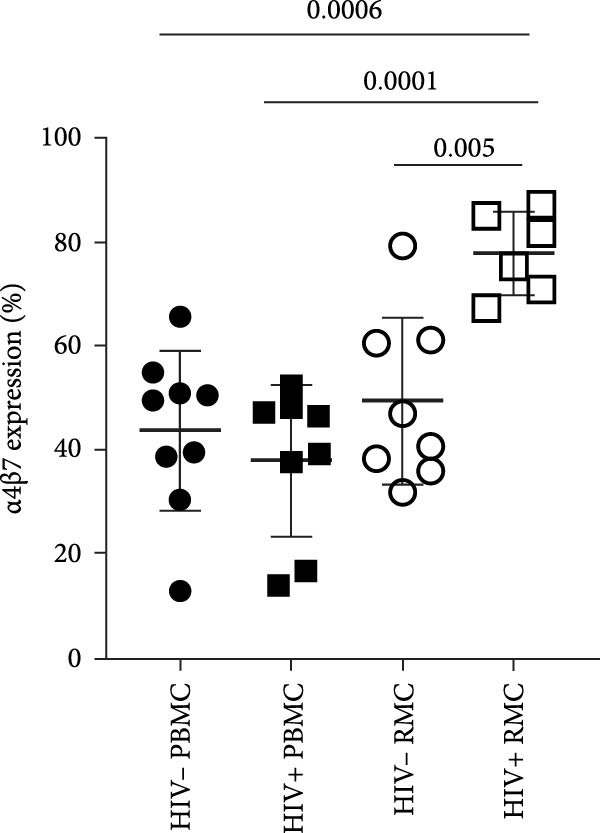
(C)

**Figure:**
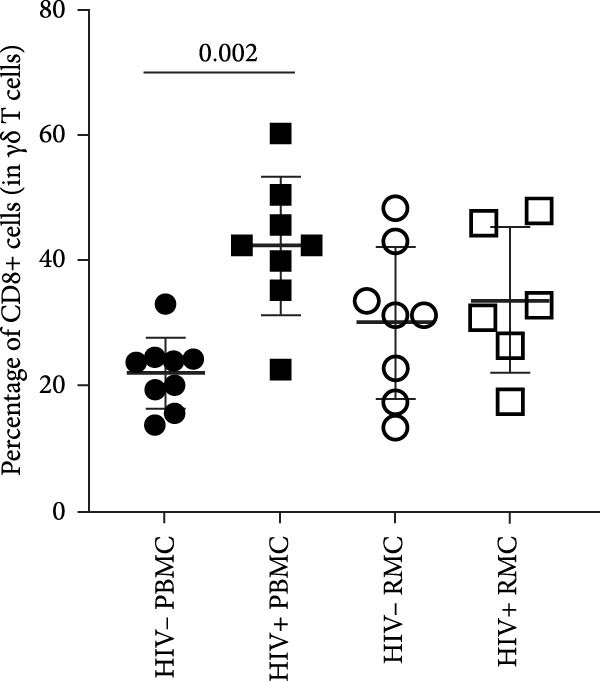
(D)

**Figure:**
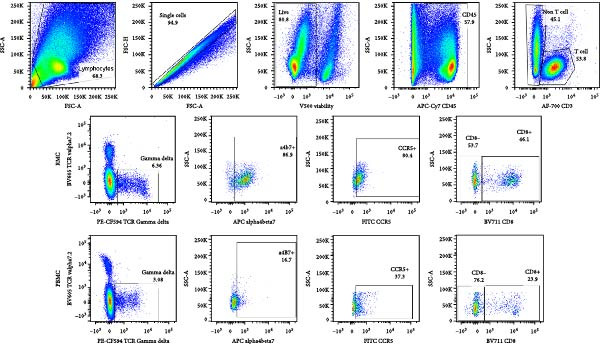
(E)

Given the potential role of CCR5 and α4β7 in the infection of γδ T cells by HIV [12], their expression was evaluated in unstimulated cells. Both PLWH and PWOH have higher proportions of CCR5+ γδ T cells in the gut as compared to peripheral blood, but no significant difference was observed between PLWH and PWOH (Figure 1B). Next, the integrin α4β7 was evaluated. Higher levels of α4β7+ γδ T cells were observed in the recto‐sigmoid colon biopsies of PLWH, compared to those observed in the blood. In PWOH, expression of α4β7 was also found to be higher in gut‐derived cells, but this difference did not meet statistical significance. Overall, α4β7 expression in gut‐derived γδ T cells of PLWH was highest (Figure 1C).

CD8‐expressing γδ T cells have been implicated in both protective and pathogenic roles [2, 19, 20]. A higher proportion of circulating CD8+ γδ T cells was observed in PLWH when compared to CD8+ γδ T cells from PWOH (Figure 1D), while no significant difference between groups was observed in the gut. Gating strategy for both PBMC and RMC and representative dot plots for γδ, CCR5, α4β7, and CD8 expression from one PLWH donor are shown (Figure 1E).

### 3.2. Single Cytokine‐Producing Bulk and CD8+ γδ T Cells

For evaluation of γδ T cell function, the expression of selected cytokines was examined in γδ T cells by intracellular cytokine staining and flow cytometry. The expression of IFN‐γ, TNF‐α, and IL‐17 was evaluated in PBMC and RMC following stimulation with PMA and Ionomycin for 5 h. The proportion of γδ T cells expressing IFN‐γ was significantly lower in gut‐derived cells as compared to their blood counterparts in both groups; however, there was a higher proportion of gut‐derived IFN‐γ+ γδ T cells from PLWH compared to PWOH (Figure 2A). Furthermore, IL‐17+ γδ T cells were present in higher proportions in the gut in both PLWH and PWOH (Figure 2B), while gut‐derived TNF‐α+ γδ T cells were observed in lower proportions when compared to the peripheral blood (Figure 2C). No significant difference was observed in the proportion of IL‐17 or TNF‐α cytokine‐producing cells between PWOH and PLWH donors.

Figure 2Cytokine expression in bulk and CD8+ γδ T cell populations in PBMC and RMC from ART‐treated PLWH and PWOH. PBMC and RMC were isolated from PWOH (HIV−) and ART‐treated PLWH (HIV+). Following stimulation with PMA/Ionomycin for 5 h, expression of IFN‐γ (bulk: A, CD8+: D), IL‐17 (bulk: B, CD8+: E), and TNF‐α (bulk: C, CD8+: F) were evaluated by flow cytometry and values obtained from stimulated cells were subtracted from unstimulated ones and are shown in the graph (PBMC: HIV− *n* = 9, HIV+ *n* = 8; RMC: HIV− *n* = 8, HIV+ *n* = 6). (G) Representative dot plots for unstimulated and stimulated RMC from one PLWH donor. Significant differences are shown in the graph, calculated by ANOVA and Tukey’s test.

**Figure:**
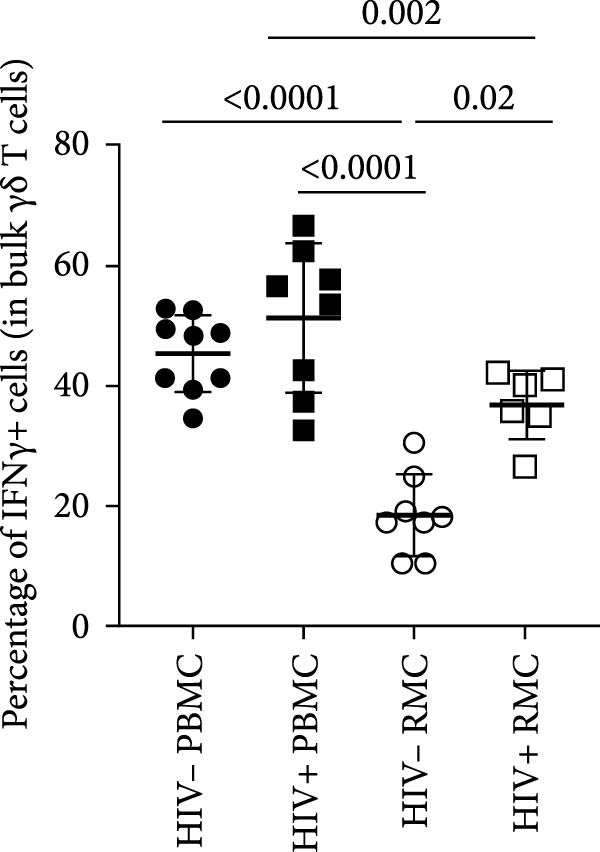
(A)

**Figure:**
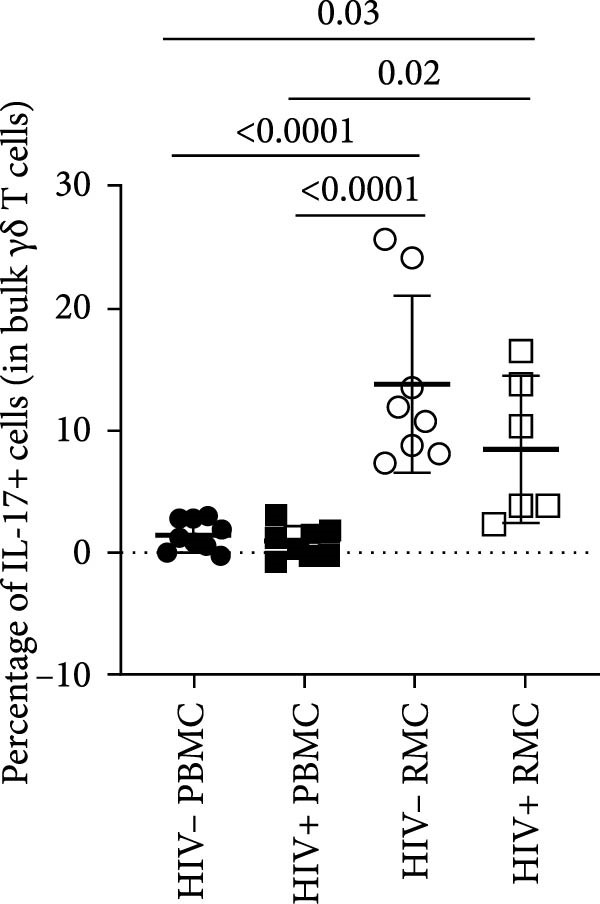
(B)

**Figure:**
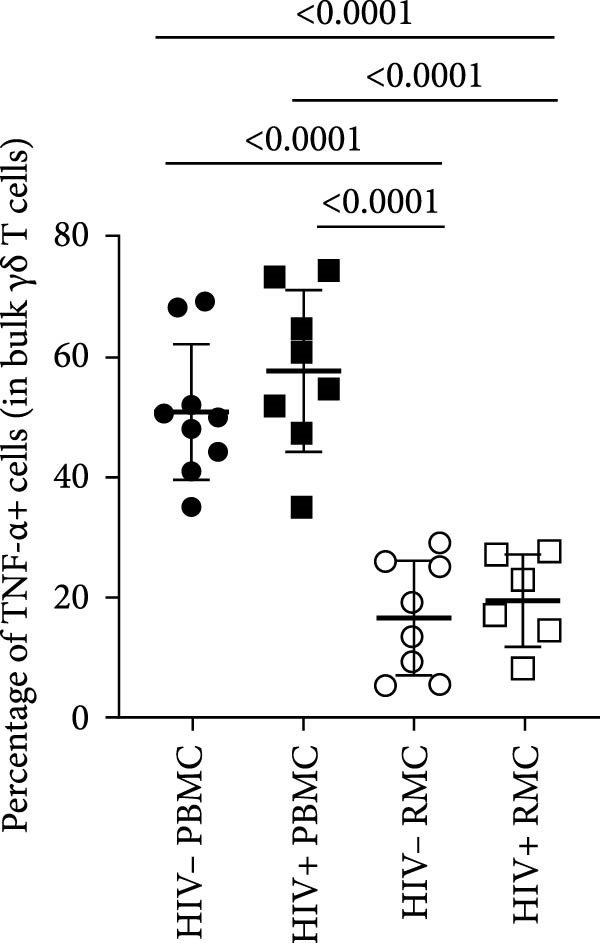
(C)

**Figure:**
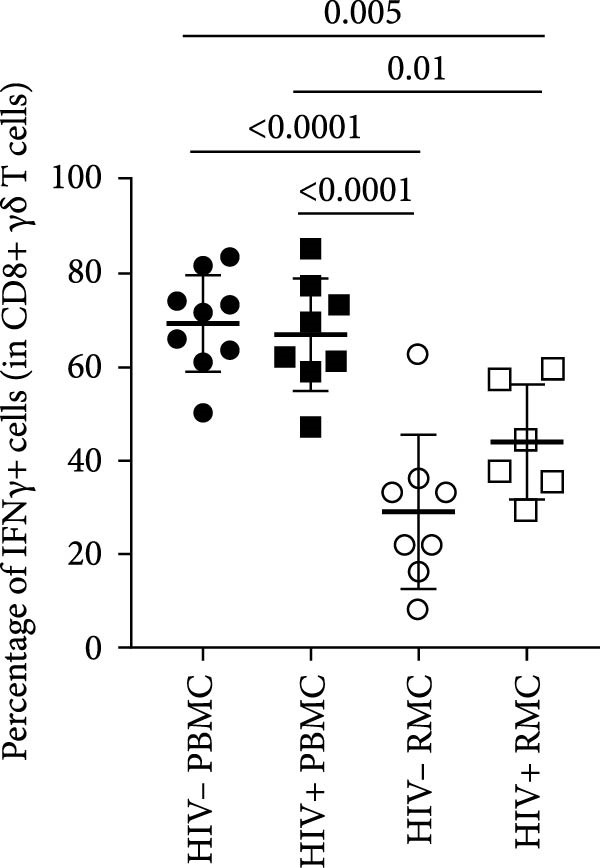
(D)

**Figure:**
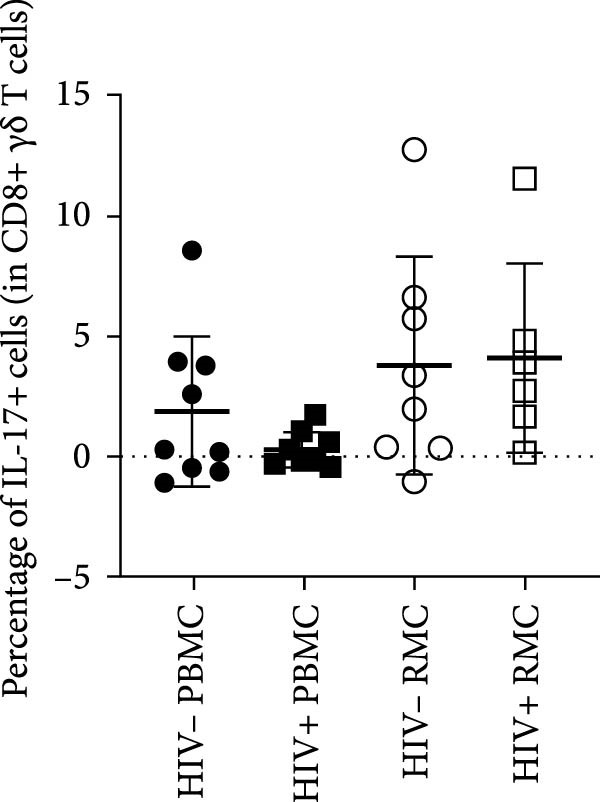
(E)

**Figure:**
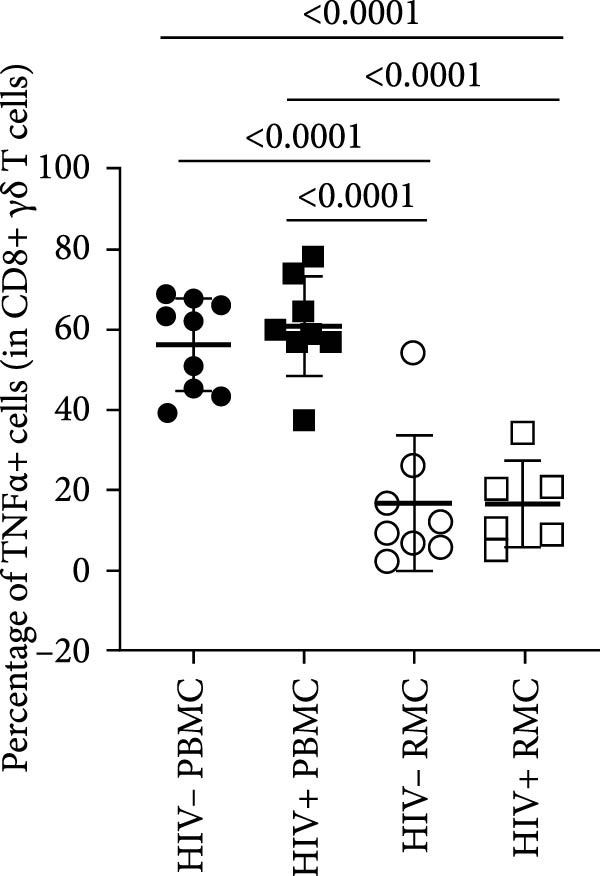
(F)

**Figure:**
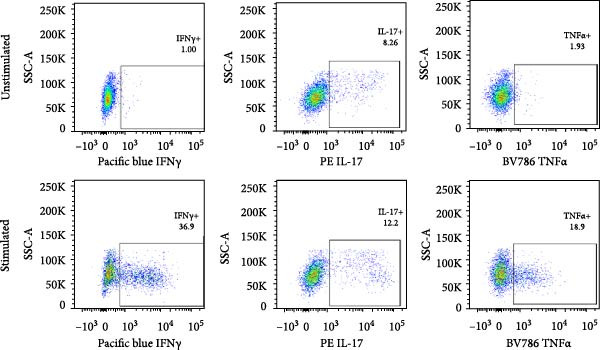
(G)

The proportions of cytokine‐expressing CD8+ γδ T cells were then evaluated in both groups. Like what was observed in bulk γδ T cells, there were fewer IFN‐γ‐expressing CD8+ γδ T cells in the gut when compared to blood in both groups, and there was a trend toward higher expression of IFN‐γ in gut‐derived cells from PLWH than PWOH (Figure 2D). No significant difference was observed in IL‐17+ CD8+ γδ T cells in either location or group (Figure 2E). As for TNF‐α‐expressing CD8+ γδ T cells, lower proportions of these cells were observed in the gut of both PWOH and PLWH when compared to the blood (Figure 2F). Representative dot plots for unstimulated and stimulated RMC from one PLWH donor are shown (Figure 2G).

### 3.3. Polyfunctionality of Bulk γδ T Cells and CD8+ γδ T Cells

**Figure 3:**
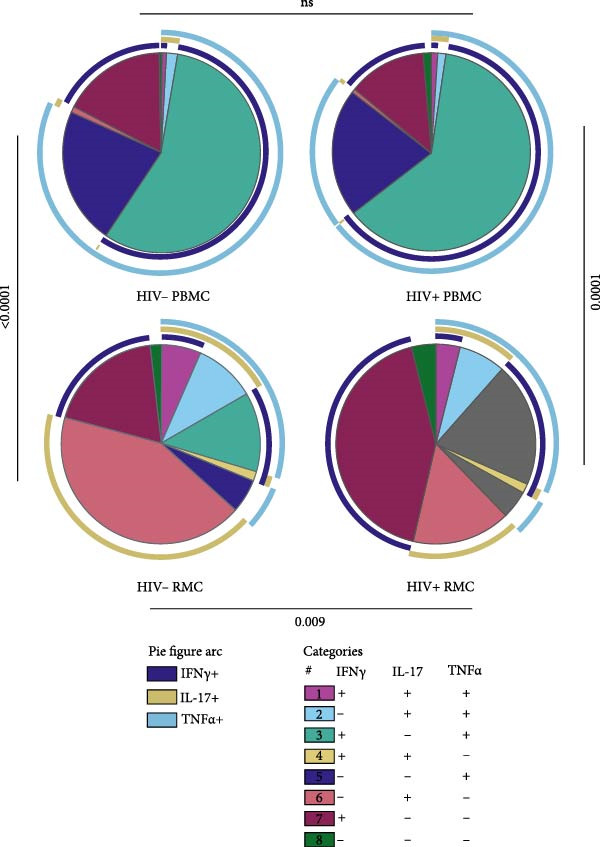
Polyfunctionality profile of bulk γδ T cells in PBMC and RMC from ART‐treated PLWH and PWOH. PBMC and RMC were isolated from PWOH and ART‐treated PLWH. Bulk γδ T cell expression of IFN‐γ, IL‐17, and TNF‐α was evaluated after stimulation with PMA/Ionomycin for 5 h and analyzed by flow cytometry. Pie charts show the proportion of cells expressing various measured cytokines as described in the Categories legend, pie arcs show the expression of each cytokine (purple: IFN‐γ, beige: IL‐17, and blue: TNF‐α) (PBMC: healthy *n* = 9, HIV *n* = 8; RMC: healthy *n* = 8, HIV *n* = 6). Significant differences are shown on the graph, calculated by NIH SPICE.

Finally, we evaluated the polyfunctionality of γδ T cell populations in the blood and gut of PLWH and PWOH. These analyses were only performed on bulk γδ T cells, given the similarity in cytokine expression when differentiating by CD8 expression. A permutation test of polyfunctionality was performed in SPICE [18]. A significant difference in the polyfunctionality profile of gut‐derived γδ T cells between PLWH and PWOH was observed (Figure 3). In PLWH, IFN‐γ single‐expressing cells make up the largest subset, followed by IFN‐γ and TNF‐α coexpressors, then IL‐17 single‐expressing cells. In PWOH, the largest population is IL‐17 single‐expressing cells, followed by IFN‐γ single‐expressors, then IFN‐γ and TNF‐α coexpressing cells. The distinct profile in gut‐derived γδ T cells from PLWH is largely due to high IFN‐γ expression, as seen in Figure 2A. It is possible that gut IL‐17+ γδ T cells are also impacted by HIV infection; however, no significant difference was observed when comparing IL‐17‐expressing γδ T cells in the gut of PLWH vs. PWOH (Figure 2B). In the blood, no significant difference was observed between cells derived from PLWH and PWOH; however, the polyfunctionality profile of blood‐derived cells was significantly different than that of gut‐derived cells in both groups, with IFN‐γ and TNF‐α coexpressing cells forming the largest population (Figure 3), and a large amount of TNF‐α and very little IL‐17 overall, as seen in Figure 2B,C.

## 4. Discussion

While the effect of HIV infection on γδ T cells in peripheral blood has been well documented, the impact of HIV on γδ T cells from the gastrointestinal tract remains largely unknown. In this study, blood‐ and recto‐sigmoid colon‐derived γδ T cells from PLWH on effective ART were evaluated in comparison to those from PWOH. Blood‐derived γδ T cells were similar with respect to CCR5 expression, and expression of IL‐17, TNF‐α, and polyfunctional profile upon stimulation, in both PLWH and PWOH. Recto‐sigmoid colon‐derived cells had a different polyfunctionality profile, higher proportions of α4β7, and higher expression of IFN‐γ upon stimulation in PLWH as compared to PWOH.

CCR5 is the main HIV coreceptor, involved in both virus entry and cell‐to‐cell spread. Higher levels of peripheral CCR5+ CD4+ T cells have been observed in untreated HIV [21, 22], and CCR5 expression is upregulated on circulating Vδ2 cells during viremia [13]. As the expression of CCR5 was found to be similarly higher in the gut in both PLWH and PWOH, it suggests that ART can normalize CCR5 expression by γδ T cells. This has been previously shown on CD4+ T cells [23, 24] and in endocervical mucosa‐derived Vδ2 T cells [9]. Given the importance of CCR5 during HIV infection of cells, and its higher expression on gut‐derived cells reported here, these data suggest that γδ T cells present in the gut may be more permissive to HIV infection than their blood counterparts.

α4β7 is a gut‐homing marker, and its expression has been associated with a higher susceptibility to HIV infection [25, 26], as α4β7 binding to gp120 in the absence of CD4 is possible due to CCR5 surface expression. This has been shown on Vδ2 T cells, where increased susceptibility to infection is implicated in HIV‐mediated depletion [12]. The expression of α4β7 was found here to be highest in recto‐sigmoid colon‐derived γδ T cells from PLWH. This greater expression may be due to the trafficking of other α4β7+ γδ T cells, such as Vδ1, to the gut, as demonstrated in SIV + macaques [27]. Regardless, elevated expression of α4β7 in gut‐derived γδ T cells might indicate a mechanism of viral pathogenesis by which the rapid spread of the infection in the gut compartment is facilitated.

In this study, PLWH had a significantly higher proportion of gut‐derived IFN‐γ+ γδ T cells as compared to PWOH. These IFN‐γ+ cells could be involved in the disruption of gut mucosal immune function that persists despite effective ART during HIV infection. In ART‐treated SIV‐infected macaques, expression of IFN‐γ was higher than prior to infection in both peripheral blood and gut‐derived γδ T cells, with enhanced TNF‐α production and reduction in IL‐17 expression, demonstrating a loss of Th17 phenotype and a trend toward a Th1 phenotype [28]. As the present study also found a higher proportion of gut‐derived γδ T cells expressing IFN‐γ in PLWH, there may also be a trend toward a Th1 phenotype in these cells despite successful ART, though no change in TNF‐α and IL‐17 was detected.

Less polyfunctionality of blood‐derived γδ T cells from PLWH has been reported [10, 11], which is of interest as polyfunctionality of HIV‐specific CD8+ T cells is associated with enhanced HIV disease control [29]. Here, it was found that blood‐derived γδ T cells from PLWH did not have differing polyfunctionality of the cytokines we evaluated compared to PWOH. However, successful ART did not restore the functional profile of gut‐derived γδ T cells to that of PWOH. This is similar to what was found in SIV‐infected macaques, where 9 months of treatment did not restore the profile of gut‐derived γδ T cell polyfunctionality to preinfection profiles [28]. All this suggests that immune recovery may be different in distinct compartments.

The inability to match PLWH and PWOH for age and sex is a limitation of this study, though the differences in ages of these two groups would not be expected to translate to a significant difference in the immune cell composition in the compartments studied [30]. Future studies should attempt to clarify how the proportions of Vδ1 and Vδ2 subsets are represented within the two locations with respect to other parameters studied here, as antibodies specific for Vδ1 and Vδ2 were not included in the flow cytometry panel used for the HAVARTI clinical trial.

## 5. Conclusion

The impact of HIV on the gut was characterized by elevated levels of both α4β7 and IFN‐γ expression by γδ T cells, along with alterations in the polyfunctionality of these cells when compared to PWOH. Taken together, our results demonstrate that despite successful long‐term ART, γδ T cell functionality remains significantly altered in the gut, while the function of their blood‐derived counterparts seems to be restored. More studies are needed to further characterize this phenomenon, particularly in the analysis of each of the main γδ T cell subsets (Vδ1 and Vδ2) and their contribution to the impaired polyfunctionality in the gut. Given the importance of γδ T cells in both innate and adaptive immune responses and regulation, it is possible that the alterations described here play a role in the ongoing systemic inflammation observed in PLWH despite long‐term successful ART.

## 6. Data Limitations and Perspectives

A limitation of this study was the inability to match PLWH and PWOH for age and sex. We are restricted in that participants in such a clinical trial (Havarti, PLWH) are rare, and we can only use what is available to us, and that PWOH undergoing colonoscopy are, by nature, older. The PWOH biopsy and blood samples were also not individually matched, as recruiting PWOH undergoing colonoscopy was a logistical challenge, and collecting matching blood samples was not part of standard care. The pinch biopsy technique used here does not allow for dissection between each specific tissue and may have included cells belonging to intestinal epithelium, lamina propria, and intestinal lymphoid follicles from the recto‐sigmoid colon, randomly. Therefore, this data cannot be used to infer the characteristics of γδ T cells present in any specific tissue in the recto‐sigmoid colon, as described previously [17].

**Nomenclature**
ART: Antiretroviral therapy
HIV: Human immunodeficiency virus
PBMCs: Peripheral blood mononuclear cells
PLWH: People living with HIV
PWOH: People without HIV
RMCs: Recto‐sigmoid mononuclear cells.

## Ethics Statement

The present study was performed with data and material obtained for the clinical trial HAVARTI which was approved by Ottawa Health Science Network Research Ethics Board (OHSN‐REB 20160928 and 2005256‐O1H) (Ottawa, ON, Canada).

## Consent

All participants provided written informed consent.

## Disclosure

This work does not involve animal studies.

## Conflicts of Interest

The authors declare no conflicts of interest.

## Author Contributions

**Priscila O. Barros**: methodology, formal analysis, investigation, writing – original draft, writing – review and editing, visualization. **Stephanie C. Burke Schinkel**: methodology, investigation, writing – review and editing, visualization. **Ameeta Lubina Nayak**: methodology, investigation, writing – review and editing. **Brittany Haas**: writing – review and editing. **Tamara K. Berthoud**: methodology, investigation, writing – review and editing. **Michaeline McGuinty**: resources, writing – review and editing. **D. William Cameron**: resources, writing – review and editing. **Jonathan B. Angel**: methodology, resources, writing – review and editing, supervision, project administration, funding acquisition.

## Funding

This study was supported by the Canadian HIV Trials Network, Canadian Institutes of Health Research; the Canadian Institute of Health Research funded Canadian HIV Cure Enterprise (CanCURE) Team (Grant HB2‐164064); and the Division of Infectious Diseases, University of Ottawa at the Ottawa Hospital.

## Data Availability

The data that support the findings of this study are available from the corresponding author upon reasonable request. No material was obtained from other sources.
